# Validation of data quality in a nationwide gastroenterological surgical database: The National Clinical Database site‐visit and remote audits, 2016‐2018

**DOI:** 10.1002/ags3.12419

**Published:** 2020-12-22

**Authors:** Hiroshi Hasegawa, Arata Takahashi, Shingo Kanaji, Yoshihiro Kakeji, Shigeru Marubashi, Hiroyuki Konno, Mitsukazu Gotoh, Hiroaki Miyata, Yuko Kitagawa, Yasuyuki Seto

**Affiliations:** ^1^ NCD Data Quality Management Subcommittee The Japanese Society of Gastroenterological Surgery Tokyo Japan; ^2^ Department of Health Policy and Management School of Medicine Keio University Tokyo Japan; ^3^ Department of Healthcare Quality Assessment Graduate School of Medicine The University of Tokyo Tokyo Japan; ^4^ Database Committee The Japanese Society of Gastroenterological Surgery Tokyo Japan; ^5^ The Japanese Society of Gastroenterological Surgery Tokyo Japan; ^6^ National Clinical Database Tokyo Japan

**Keywords:** audit, gastroenterological surgery, National Clinical Database

## Abstract

**Background and Aim:**

In 2015, the Japanese Society of Gastroenterological Surgery (JSGS) initiated data verification in the gastroenterological section of the National Clinical Database (NCD) and reported high accuracy of data entry. Remote audits were introduced for data validation on a trial basis in 2016 and formally accepted into use in 2017‐2018. The aim of this study was to audit the data quality of the NCD gastroenterological section for 2016‐2018 and to confirm the high accuracy of data in remote audits.

**Methods:**

Each year, 45‐46 hospitals were selected for audit. Twenty cases were randomly selected in each hospital, and the accuracy of patient demographic and surgical outcome data (46 items) was compared with the corresponding medical records obtained by visiting the hospital (site‐visit audit) or by mailing data from the hospital to the JSGS office (remote audit).

**Results:**

A total of 136 hospitals were included, of which 88 (64.7%) had a remote audit, and 124 936 items were evaluated with an overall data accuracy of 98.1%. There was no significant difference in terms of data accuracy between site‐visit audit and remote audit. Accuracy, sensitivity, and specificity of mortality were 99.7%, 89.7%, 100% for site‐visit audits and 99.8%, 97.3%, 100% for remote audits, respectively. Mean time spent on data verification per case of remote audits was shorter than that of site‐visit audits (10.0 minutes vs 13.7 minutes, *P* < 0.001).

**Conclusion:**

The audits showed that NCD data are reliable and characterized by high accuracy. Remote audits may substitute site‐visit audits.

## Graphical abstract

The Japanese National Clinical Database (NCD) covers > 95% of the surgical operations carried out in Japan. The aim of this study was to audit the data quality of the NCD gastroenterological section for 2016‐2018. The audits showed that NCD data are reliable and characterized by high accuracy.

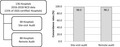

## 1 INTRODUCTION

The Japanese National Clinical Database (NCD) is a large‐scale, nationwide, web‐based data entry system linked to the surgical board certification system and covering >95% of the surgical operations carried out in Japan.^1^ The goal of the NCD is to provide a basis for the best surgical treatment for the citizens and maintain the high standard of surgical quality in Japan. This is done through systematic gathering and analysis of clinical data serving to inform advances in quality improvement.

In the gastroenterological section of the NCD, the Japanese Society of Gastroenterological Surgery (JSGS) selected 115 gastroenterological operative procedures as important for surgical training in the board certification system. From 2011 to 2018, a total of 4 420 168 gastroenterological surgical cases were recorded.^2^, ^3^, ^4^ The JSGS also selected eight main procedures (esophagectomy, distal gastrectomy, total gastrectomy, right hemicolectomy, low anterior resection, hepatectomy, pancreaticoduodenectomy, and surgery for acute diffuse peritonitis) as particularly important as medical standards for improving surgical quality. Risk models of mortality and morbidity for these eight main procedures have been developed, and a risk calculator has been implemented using these risk models, which enables predictions of patient morbidity and mortality upon entering of the preoperative data; this calculator is available on the NCD website for physicians in clinical practice.

The JSGS began its data verification activity in 2015, focusing on NCD data representing these eight main procedures and found a high accuracy of data entry.^5^ In this initial audit, 17 hospitals (2% of the JSGS‐certified hospitals) were selected, and data from all relevant medical records were obtained by visiting the hospitals (site‐visit audit). Later, in 2017, the JSGS initiated a full‐scale audit: Each year, 45‐46 hospitals (5% of the JSGS‐certified hospitals) were selected for audits. In addition to site‐visit audits, the JSGS introduced the remote audit as an alternative to the site‐visit audit; in the remote audit, data from relevant medical records were obtained by mailing the data from the hospital to the JSGS office. In the present study, we document the NCD data verification activity for the gastroenterological section for the period of 2016‐2018.

## 2 METHODS

### 2.1 Data sources

The NCD was established in 2010 as a general incorporated association in collaboration with several clinical societies. In the gastroenterological section of the NCD, the JSGS selected 115 gastroenterological operative procedures for the board certification system. These 115 procedures were stipulated by the Training Curriculum for Board Certified Surgeons in Gastroenterology. The JSGS selected eight main procedures (esophagectomy, distal gastrectomy, total gastrectomy, right hemicolectomy, low anterior resection, hepatectomy, pancreaticoduodenectomy, and surgery for acute diffuse peritonitis) as particularly important in terms of medical standards for surgical quality assessment. As previously reported,^5^ the subjects were patients whose surgical data were recorded in the NCD and who underwent one or more of the eight main procedures. JSGS‐certified hospitals were requested to register their gastroenterological surgery cases in the NCD. Data were collected using specialized web‐based data collection forms, which contained approximately 250 variables, including data on demographics, preoperative risk, operative procedure, postoperative complications, and outcomes. Registration was closed annually on a fixed date to block further entries. Each participating hospital was requested to appoint a data manager to be accountable for data traceability. The protocol for the NCD project, which included a site‐visit audit, was approved by the Japan Surgical Society and the institutional ethics board of Tokyo University (registry number: 2976). In order to be able to enter patient data into the database, each participating hospital was requested to obtain ethical approval from its local institutional review board, the facility director's permission, or ethical approval from a proxy review board. Remote audits were approved by JSGS and the institutional ethics board of Kobe University (registry number: 170169). For remote audits, each participating hospital required permission from the facility director or ethical approval from the local institutional review board.

### 2.2 Methods of data verification

After the test audit (data on surgical cases pertaining to the year 2014 in 13 hospitals) carried out in 2015 and the initial audit (data on surgical cases pertaining to the year 2015 in 17 hospitals) in 2016, 45‐46 hospitals (5% of the JSGS‐certified hospitals) were selected for auditing each year. As previously reported,^5^ to confirm the feasibility of data verification, 20 cases were randomly selected from each hospital to ensure a sufficient number of cases for comparison.

Medical records were obtained by visiting the hospital (site‐visit audit) or by mailing data from the hospital to the JSGS office (remote audit). Remote audits were introduced for data validation on a trial basis in 2016 and formally taken into use in 2017‐2018. Each hospital decided on which audit to choose. For the remote audit, the JSGS mailed the registration number on each of the 20 cases, and each hospital mailed the copy of the corresponding medical records to JSGS office. The medical record includes a discharge summary and holds information regarding operation(s), anesthesia, and hospitalization. To protect sensitive personal information, anonymization was undertaken by each hospital in a linkable manner, and a secure mail service was used.

Accuracy assessment involved variables, such as patient demographics, intraoperative information, and outcomes. We established a protocol to evaluate these variables in the test and initial audits. We defined the priority of checking the source documents and developed a flow chart to judge each variable. For example, surgical profile (gender, hospital admission data, preoperative diagnosis, postoperative diagnosis, and hospital discharge data) was checked and judged by the discharge summary. If the data was judged to be imprecise, the next step was to check the operation record, doctor's record, and nurse's record. Operative data (operation date, operator and assistant, surgical procedure, wound class, anesthesia technique, intraoperative blood loss, and operative time) was checked and judged by operation record and anesthesia record. Identification of the variables in the existing medical records was relatively straight‐forward, and the definitions of the terms were unambiguous because they were standardized. During each remote audit, we referred to the medical record mailed by each hospital and evaluated the consistency of the variable values with those in the registered data. If the data submitted to the NCD matched those in the source documents, we judged the items as “consistent.” Meanwhile, in cases of inconsistent values/information, we sought additional information to identify the cause of the discrepancy and requested additional material from the hospital.

Two or three staff members who were independent of the JGSG, not involved in any clinical practice, and who had general medical knowledge (nurse or health information manager) performed the established audit according to protocol under the supervision of a medical doctor belonging to the quality management subcommittee of the JGSG. All auditors were required to sign a written contract in which they consented to adhering strictly to the confidentiality obligations with regard to the hospital information and they were allowed access to the data for the purpose of verification only.

The feedback system followed an established process. All discordant results were provided to each hospital and were corrected. All audit results were analyzed by members of the JSGS database committee and the NCD data quality management subcommittee. A general outline of audit results and solutions to improve data quality was provided to all members of JSGS.

We measured the total audit time required for each facility. The time spent for data verification per case was calculated by dividing the total audit time by the number of audit cases.

### 2.3 Statistical analyses

The accuracy of data entry was expressed as the proportion of consistent items per verified case. We also calculated an item‐wise proportion of data consistency between the source data and the NCD data. Some items, for which the original source could not be identified by the unified method, were considered indeterminable and excluded from analysis. We calculated the sensitivity, specificity, positive predictive value (PPV), and negative predictive value (NPV) for complications, transfusions, and mortality measures at discharge and 30 days after surgery. The time taken to verify the data for each case was compared by type of audit. All statistical analyses were carried out using the JMP v.14.0 software (SAS Institute Inc., Cary, NC, USA).

## 3 RESULTS

A total of 136 hospitals were selected for data validation (Table 1), of which 88 (64.7%) had a remote audit. Remote audit was introduced for data validation on a trial basis (3 hospitals) in 2016 and was formally used in the 2017‐2018 data validation. Remote audits were used for 42 hospitals (91.3%) in the 2017 data validation and 43 hospitals (95.6%) in the 2018 data validation.

**Table 1:** Type of audit used to evaluate data quality of the NCD gastroenterological section for 2016‐2018

Type of audit (no. of hospitals)	Total	2016	2017	2018
Site‐visit audit	48	42	4	2
Remote audit	88	3	42	43

### 3.1 Accuracy of data entry

The total number of cases selected for the 2016‐2018 audits was 2716, and 124 936 items were assessed. Table 2 provides an overview of the concordance rate for site‐visit audits and remote audits. The overall concordance rate of remote audits was slightly superior than that of site‐visit audits, without the difference reaching statistical significance (remote audit concordance, 98.2%; site‐visit audit concordance, 98.0%; *P* = 0.051). Most data accuracies were >95%; however, the accuracy of data on wound class, weight, anesthesia technique, and American Society of Anesthesiologists (ASA) class was <95% in both site‐visit audits and remote audits. Data accuracy for all postoperative occurrences were >95%. Measures of mortality at discharge and 30 days after surgery showed an agreement of >99%.

**Table 2:** Concordance rates for site‐visit audits and remote audits

Variable	Concordance rates (%)			*P* *
	Total	Site‐visit audit	Remote audit	
Audited variables (n)	124 936	44 160	80 776	
Overall concordance	98.1	98.0	98.2	0.051
Surgical profile				
Date of birth	99.2	99.1	99.2	0.703
Gender	99.9	99.7	99.9	0.097
Hospital admission date	97.0	96.8	97.1	0.636
Preoperative diagnosis	99.0	99.2	99.0	0.624
Postoperative diagnosis	98.6	98.8	98.6	0.709
Hospital discharge date	97.6	97.1	97.8	0.223
Preoperative risk factors				
Pack‐year cigarette history	95.9	95.3	96.3	0.213
History of severe COPD	98.7	99.4	98.4	0.030
Current dialysis	99.9	100.0	99.9	0.296
Sepsis	99.8	99.9	99.7	0.338
Height	98.1	97.1	98.6	0.005
Weight	89.3	87.9	90.0	0.088
Operative data				
Emergency case	99.4	99.8	99.2	0.055
Nature of the tumor	99.1	99.6	98.9	0.055
Location of malignant tumor	99.1	99.4	98.9	0.233
Operation date	99.2	98.9	99.3	0.209
Operator and assistant	95.8	97.1	95.2	0.016
Surgical procedure	99.0	99.7	98.6	0.008
Wound class	92.7	90.7	93.8	0.003
Cardiac arrest requiring CPR	99.9	100.0	99.9	0.296
Anesthesia technique	90.5	91.1	90.1	0.371
Intraoperative blood loss	96.2	96.5	96.1	0.665
Operative time	95.7	93.3	97.0	<0.001
ASA class	87.2	86.5	87.6	0.377
Postoperative occurrences				
Superficial incisional SSI	98.8	98.9	98.8	0.908
Deep incisional SSI	99.6	99.4	99.7	0.182
Organ/space SSI	96.9	96.5	97.2	0.318
Wound disruption	100.0	100.0	100.0	1
Pneumonia	99.1	99.1	99.1	0.825
Pulmonary embolism	100.0	100.0	100.0	1
Ventilated for >48 h	99.7	99.8	99.6	0.409
Urinary tract infection	99.5	99.1	99.8	0.010
Vein thrombosis	99.8	100.0	99.7	0.098
Sepsis/septic shock	99.7	99.6	99.7	0.758
Unexpected intubation	99.6	99.5	99.7	0.331
Renal insufficiency	99.7	99.9	99.7	0.243
Stroke/CVA	100.0	100.0	100.0	1
Coma	100.0	100.0	100.0	1
Peripheral nerve injury	100.0	100.0	100.0	1
Arrest	100.0	100.0	100.0	1
Intra‐/postoperative MI	100.0	100.0	100.0	1
Intra‐/postoperative Transfusion	98.2	98.1	98.2	0.837
Discharge data				
Mortality	99.8	99.7	99.8	0.452
30‐d follow up	99.4	99.5	99.4	0.731
Readmission	98.8	99.0	98.7	0.626
Unplanned reoperation	98.9	99.4	98.6	0.077

Abbreviations: ASA, American Society of Anesthesiologists; COPD, chronic obstructive pulmonary disease; CPR, cardio‐pulmonary resuscitation; CVA, cerebrovascular accident; MI, myocardial infarction; SSI, surgical site infection.

* *P* Site‐visit audit vs Remote audit.

### 3.2 Accuracy statistics for mortality and complications

Table 3 displays the sensitivity, specificity, PPV, and NPV of postoperative complications and mortality as compared with medical record reviews. Measures of mortality at discharge showed a 99.8% agreement rate, with a sensitivity of 93.9% and specificity of 99.9%. Among the six complications studied, organ/space surgical site infection (SSI) was the most frequent. While specificity was constantly high throughout the whole variable (>98.9%), the sensitivity of organ/space SSI was relatively low (71.1% overall, 74.4% in site‐visit audits, and 68.5% in remote audits).

**Table 3:** Reliability of mortality and complication records as compared with reviews of medical records

	Concordant		Discordant		Sensitivity	Specificity	PPV	NPV
	NCD: YES MR: YES	NCD: NO MR: NO	NCD: YES MR: NO	NCD: NO MR: YES				
Total								
Superficial incisional SSI	140	2541	4	28	0.833	0.998	0.972	0.989
Deep incisional SSI	66	2640	1	10	0.868	1.000	0.985	0.996
Organ/space SSI	192	2441	3	78	0.711	0.999	0.985	0.969
Pneumonia	92	2598	8	17	0.844	0.997	0.920	0.993
Sepsis/septic shock	94	2613	6	4	0.959	0.998	0.940	0.998
Intra‐/postoperative transfusion	118	2540	25	21	0.849	0.990	0.825	0.992
Mortality	62	2649	2	4	0.939	0.999	0.969	0.998
30‐d follow up	28	2549	5	0	1.000	0.998	0.848	1.000
Site‐visit audit								
Superficial incisional SSI	52	897	0	11	0.825	1.000	1.000	0.988
Deep incisional SSI	28	926	0	6	0.824	1.000	1.000	0.994
Organ/space SSI	90	838	1	31	0.744	0.999	0.989	0.964
Pneumonia	42	908	2	8	0.840	0.998	0.955	0.991
Sepsis/septic shock	34	922	3	1	0.971	0.997	0.919	0.999
Intra‐/postoperative transfusion	38	903	6	13	0.754	0.993	0.864	0.996
Mortality	26	931	0	3	0.897	1.000	1.000	0.997
30‐d follow up	9	943	2	0	1.000	0.998	0.818	1.000
Remote audit								
Superficial incisional SSI	88	1644	4	17	0.838	0.998	0.957	0.990
Deep incisional SSI	38	1714	1	4	0.905	0.999	0.974	0.998
Organ/space SSI	102	1603	2	47	0.685	0.999	0.981	0.972
Pneumonia	50	1690	6	9	0.847	0.996	0.893	0.995
Sepsis/septic shock	60	1691	3	3	0.952	0.989	0.808	0.995
Intra‐/postoperative transfusion	80	1637	19	8	0.909	0.989	0.808	0.995
Mortality	36	1718	2	1	0.973	1.000	0.947	0.999
30‐d follow up	19	1606	3	0	1.000	0.998	0.648	1.000

Abbreviations: MR, medical records; NCD, National Clinical Database; NPV, negative predictive value; PPV, positive predictive value; SSI, surgical site infection.

### 3.3 Time spent on data verification

Table 4 provides an overview of the time spent on data verification. The mean time spent for data verification per case in remote audits was shorter than that used for site‐visit audit (remote audit, 10.0 minutes; site‐visit audit, 13.7 minutes; *P* < 0.001).

**Table 4:** Time spent on data verification (min/case) in site‐visit audits and remote audits

	Site‐visit audit		Remote audit		*P*
	No. of hospitals/cases	Time (min), mean ± SD	No. of hospitals/cases	Time (min), mean ± SD	
Total	48/960	13.7 ± 4.9	88/1756	10.0 ± 3.6	<0.001
2016	42/840	13.9 ± 4.9	3/60	11.2 ± 1.6	
2017	4/80	12.2 ± 3.7	42/839	10.9 ± 4.0	
2018	2/40	11.3 ± 7.2	43/857	9.0 ± 3.1	

Abbreviation: SD, standard deviation.

## 4 DISCUSSION

Data verification provides information on completeness and accuracy of data used for investigations aiming to improve clinical practice. In this study, we provide the results of the 2016‐2018 gastroenterological section of the NCD data audit. We identified high data accuracy, with an overall concordance rate of 98.1%. This concordance rate was almost equivalent to the rate of our initial audit (98.3% in 2015)^5^ and the audit of American College of Surgeons National Surgical Quality Improvement Program (NSQIP) (96.8%–98.4% in 2005‐2008).^6^

Mortality is one of the most important surgical outcomes, and mortality risk models have been developed using the NCD data.^7^, ^8^, ^9^, ^10^, ^11^, ^12^, ^13^, ^14^ In the present study, measures of mortality at discharge showed a 99.8% agreement rate, with a sensitivity of 93.9% and a specificity of 99.9%. We only identified six cases with data disagreement on mortality at discharge; all six cases of disagreement appeared attributable to simple data entry errors. This result indicates that the NCD data are reliable and characterized by high quality, and that the mortality risk models developed so far are also reliable.

However, there is still room for improvement. The concordance rates of four individual variables were <95%. Although the concordance rate for body weight data was improved in comparison with the previous report (85.8% in the 2015 NCD data), it was still only 89.3% in 2016‐2018 NCD data. The disagreement may be caused by lack of awareness about the input item of body weight. From the NCD database view point, body weight is one of the preoperative risk items, and auditors obtained data on body weight from anesthesia records. NCD users usually enter data 90 days after surgery to provide data on 90‐day mortality, and weight loss often occurs at the time of data entry, especially after esophagectomy, gastrectomy, and pancreaticoduodenectomy.^15^, ^16^, ^17^ The concordance rates for data on wound class (92.7%), ASA class (87.2%), and anesthesia technique (90.5%) were also <95%. To reduce the number of disagreements, we have added several alarms on the NCD web input form from 2016.

There were several variables in which the concordance rates significantly differed (*P* < 0.05) between the site‐visit and remote audits. All differences in the concordance rates were small (within 4%). Remote audits had better concordance rates in height, wound class, operative time, and urinary tract infection. Data quality improvement could have contributed to these differences because most remote audits were performed at a later time. Site‐visit audits had better concordant rates in history of severe COPD, operator and assistant, and surgical procedure. The cause of these differences could not be clearly explained. Hospital selection may have contributed to these differences because the concordance rates of respective hospitals were not stable.

The cost of the audits is of major concern, because the NCD covers all parts of Japan. Although site‐visit audits are superior to remote audits in terms of the security of handling personal information, site‐visit audits entail traveling. Remote audits were introduced for data validation on a trial basis in 2016 and formally taken into use for the 2017‐2018 NCD data validation. In remote audits, all copies of the medical records and the NCD data were collated in the JSGS office. The mean time spent on data verification per case of remote audits was shorter than that of site‐visit audits. Remote audits might be superior to site‐visit audits in terms of cost.

We used a secure mail system for the remote audit as various electronic medical record systems available in Japan have compatibility problems or are simply not available online. Moreover, the penetration rate of electric medical records is not very high in Japan (46.7% in general hospitals, 85.4% in hospitals where the number of beds is >400 in 2017).^18^ All audits of the NSQIP were conducted remotely by online communication by a trained surgical and clinical reviewer.^6^ Standardization and promotion of electronic medical record systems might improve our remote audit system and quality of the NCD database.

Ideally, all hospitals should be evaluated, but the cost of the audits was prohibitive. The audit system of NCD was constructed according to that of NSQIP. A total of 45‐46 (or 5%) of JSGS‐certified hospitals were randomly evaluated each year. The difference in the average concordance rate between selected hospitals was small (not higher than 6.5%). There were only 6 hospitals (4.4% of the selected hospitals) in which the concordance rate was 95% or less. The results of audits in these years should be reflected in future audits to improve data quality.

We did not have a systematic reviewer training system, whereas the NSQIP had an online reviewer training system and examination.^6^ All audits were performed under the supervision of a medical doctor who was a member of the quality management subcommittee of the JGSG. The members of the NCD data quality management subcommittee were familiar with the NCD database and the protocol of site‐visit and remote audits. The results of audits were discussed and confirmed at the NCD periodical database meeting, and the inter‐rater reliability was guaranteed.

In conclusion, this study showed that NCD data are reliable and characterized by high accuracy. Data verification was possible by both site‐visit audit and remote audit.

## DISCLOSURE

Conflicts of Interest: AT and HM are affiliated with the Department of Healthcare Quality Assessment at The University of Tokyo. The department is a social collaboration department supported by the National Clinical Database, Johnson & Johnson KK, and Nipro Corporation. The other authors declare no conflicts of interest for this article.
